# Central hepatectomy for hepatocellular carcinoma in a patient with anti-Gerbich antibody

**DOI:** 10.1186/s40792-020-00898-7

**Published:** 2020-06-12

**Authors:** Teruo Komokata, Maki Inoue, Bibek Aryal, Hiroto Yasumura, Chinami Mori, Mituharu Nomoto, Mamoru Kaieda, Shuichi Hanada

**Affiliations:** 1 Department of Surgery, National Hospital Organization Kagoshima Medical Center, Kagoshima, Japan; 2 Department of Clinical Laboratory, National Hospital Organization Kagoshima Medical Center, Kagoshima, Japan; 3 Department of Hematology, National Hospital Organization Kagoshima Medical Center, Kagoshima, Japan

**Keywords:** Anti-Gerbich Antibody, Central hepatectomy, Preoperative autologous donation, Acute normovolemic hemodilution, Hepatocellular carcinoma

## Abstract

**Background:**

Anti-Gerbich (Ge) alloantibody against high-frequency erythrocyte antigen is extremely rare. Owing to incomplete evidence regarding the degree and severity of adverse events induced by hemolytic transfusion reactions, the transfusion management often remains cumbersome in these patients. We report an anti-Ge alloantibody positive patient with hepatocellular carcinoma (HCC) who underwent central hepatectomy (CH) without the need for an allogeneic blood transfusion.

**Case presentation:**

A 76-year-old Japanese woman was diagnosed with HCC measuring 9.5 × 8.0 cm in segments 4, 5, and 8 of the liver. This patient with anti-Ge alloantibody had a history of two pregnancies without transfusion. CH was planned, and based on the suggestion from the multidisciplinary team meeting, preoperative autologous donation (PAD) and acute normovolemic hemodilution (ANH) were performed. CH was successfully performed by using CUSA and Thunderbeat® with Pringle maneuver and infra-hepatic inferior vena cava clamping without perioperative need for an allogeneic blood transfusion. She has been alive without recurrence after a follow-up period of 45 months.

**Conclusion:**

To our knowledge, this is the first case report of hepatectomy in a patient with anti-Ge alloantibody. A multidisciplinary team approach, PAD and ANH, and bloodless liver surgical techniques appear to be useful for major hepatectomy in patients with extremely rare blood type.

## Background

Gerbich (Ge) is known to be a high-prevalence erythrocyte antigen that is present in more than 99% of the population [1]. Transfusion management in patients with positive anti-Ge alloantibody is complex in a surgical setting often requiring blood transfusion. A diverse clinical significance of anti-Ge alloantibody has been described; however, the degree and severity of adverse events induced by hemolytic transfusion reactions (HTRs) after incompatible transfusion are not precisely clear in the published literature [2].

Central hepatectomy (CH) is one of the technically challenging hepatectomies that is classified as a major liver resection based on Couinaud’s classification, and is also described as a high complexity liver resection [3]. Given the anatomical proximity of lesions to major hepatic veins, presence of two transection planes and larger exposed raw surface area, CH appears to be associated with increased risk of intra-operative blood loss and blood transfusion ratio [4, 5].

We describe an anti-Ge alloantibody positive patient with hepatocellular carcinoma (HCC) who had a successful central hepatectomy without the need of an allogeneic blood transfusion.

## Case presentation

**Fig. 1:**
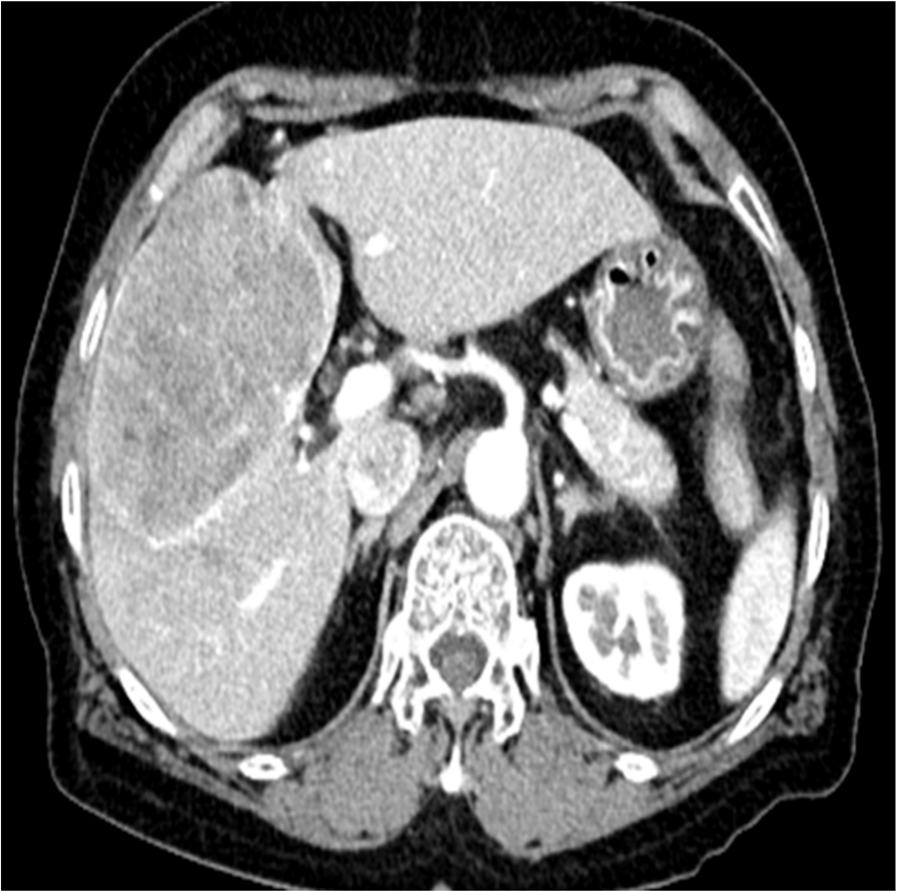
Computed tomography (CT) demonstrating a large tumor (9.5 cm in diameter) in the medial and anterior segments of the liver

A 76-year-old Japanese woman was referred to our department for evaluation of a mass in the middle section of the liver. She had no subjective symptoms and a mass was incidentally identified by an ultrasonography on her routine medical check-up. Her medical history included moderate to severe aortic stenosis, atrial fibrillation under warfarin, and mild chronic kidney disease. On presentation, her hemoglobin (Hb) level was 11.5 mg/dL, hematocrit (Ht) was 36%, and platelet count was 199,000/μl. Her total bilirubin was 0.6 mg/dl, aspartate aminotransferase (AST) was 59 U/l. alanine aminotransferase (ALT) was 19 U/l, albumin was 4.08 g/dl, and prothrombin time (PT) was 51% (INR 1.54) with warfarin use. A computed tomography (CT) scan and a magnetic resonance image (MRI) demonstrated a large tumor of 9.5 cm in diameter in the segment 4 (S4), 5 (S5), and 8 (S8) of the liver (Fig. 1). The serum alpha-fetoprotein (AFP) level was 54 ng/ml and protein induced by vitamin K absence or antagonist II (PIVKA-II) level was 210,000 mAU/ml. Hepatitis C virus antibody was positive and hepatitis B virus antigen was negative. She denied history of alcohol intake. Her Child-Pugh grade was corresponding to A with a score of point six and indocyanine green retention rate at 15 minutes was 15.8%. With the diagnosis of hepatitis C virus (HCV)-induced hepatocellular carcinoma, CH without caudate lobectomy was planned. The remnant liver volume (RLV) was estimated to be 633 mL on a three-dimensional volume analyzer (SYNAPSE VINCENT; FUJIFILM Medical Co., Tokyo, Japan). The standard liver volume (SLV) was estimated to be 942 mL by the Urata formula [6]. The RLV/SLV ratio was estimated to be 67%.

She had a history of two pregnancies with no history of blood transfusion. She was group B and RhD-positive as classified by ABO and Rh respectively. A preoperative antibody test using a polyethylene glycol/Coombs showed 3+ reactivity with all 11 panel cells (Table 1). However, additional identification test using an enzyme card revealed no reaction. The autocontrol and direct antiglobulin test were negative. Suspecting the existence of an antibody to a high-prevalence antigen, further tests on erythrocyte antigens and antibodies were requested from the reference laboratory (the Kyusyu Blood Center of the Japanese Red Cross Society in Fukuoka, Japan) where the anti-Ge antibody with titer of 32 was detected. Negative results for Ge antigen were also shown using sera including anti-Ge. Results from additional blood type antigen test other than those for the Ge antigens were as follows: C+c+E+e+, M+N+, S−s+, Le(a−b−), P_2_, Fy(a+b−), Jk(a−b+), Di(a−b+), Xg(a+), Jr(a+). There was neither frozen blood stored nor Ge negative donor registered in Japan. There were no other Ge negative members in her family.

**Table 1:** Result of antibody screening

		Rh-hr								KELL						DUFFY		KIDD		Xg	LEWIS		MNS				P	Lutheran		Results
Cell #	Rh-hr	D	C	E	c	e	f	C^W^	V	K	k	Kp^a^	Kp^b^	Js^a^	Js^b^	Fy^a^	Fy^b^	Jk^a^	Jk^b^	Xg^a^	Le^a^	Le^b^	S	s	M	N	P_1_	Lu^a^	Lu^b^	PEG/ Coombs
1	R1wR1	+	+	0	0	+	0	+	0	0	+	0	+	0	+	+	0	+	+	+	0	+	0	+	+	+	+	0	+	3+
2	R1R1	+	+	0	0	+	0	0	0	+	+	0	+	nt	+	0	+	+	0	0	+	0	+	+	+	+	+	0	+	3+
3	R2R2	+	0	+	+	0	0	0	0	+	+	0	+	nt	+	+	+	0	+	+	0	0	0	+	+	0	+	0	+	3+
4	R_0_r	+	0	0	+	+	+	0	+	0	+	0	+	0	+	0	0	+	0	0	0	0	+	+	+	+	+	0	+	3+
5	r’r	0	+	0	+	+	+	0	0	0	+	0	+	0	+	+	+	+	+	0	+	0	+	+	+	0	+	0	+	3+
6	r”r	0	0	+	+	+	+	0	0	0	+	0	+	0	+	0	+	0	+	+	0	+	+	+	+	+	+	0	+	3+
7	rr	0	0	0	+	+	+	0	0	+	+	+	+	0	+	0	+	+	+	+	0	+	+	+	+	+	+	0	+	3+
8	rr	0	0	0	+	+	+	0	0	0	+	0	+	nt	+	0	+	+	0	+	+	0	0	+	0	+	+	0	+	3+
9	rr	0	0	0	+	+	+	0	0	0	+	0	+	nt	+	+	+	0	+	+	0	+	+	0	+	0	0	0	+	3+
10	rr	0	0	0	+	+	+	0	0	0	+	0	+	0	+	+	0	+	+	0	0	+	0	+	0	+	0	0	+	3+
11	R1R1	+	+	0	0	+	0	0	0	0	+	0	+	nt	+	0	+	0	+	+	0	+	+	0	+	0	+	+	+	3+
	Autocontrol																													0

*nt* not tested*, PEG* polyethylene glycol

The management of the patient was intensively discussed with a multidisciplinary team of experts from the departments of hematology, clinical laboratory, oncology, hepatology, radiology, and anesthesiology. Since it was hard to predict the degree and severity of adverse events related to HTRs by incompatible transfusion, preoperative autologous donation (PAD) and acute normovolemic hemodilution (ANH) were planned to avoid perioperative allogenic blood transfusion as far as possible. After explaining the risks and benefits of the surgical intervention with the possibility of incompatible transfusion to the patient, she agreed to proceed for the surgery. A total of 800 ml autologous blood was preserved preoperatively under erythropoietin therapy, epoetin beta 6000 IU intravenous administration three times a week, supplemented by the daily administration of iron. Warfarin was interrupted 4 days before surgery, subsequently intravenous unfractionated heparin was started at 10,000 units per day, and stopped 6 h before surgery.

**Fig. 2:**
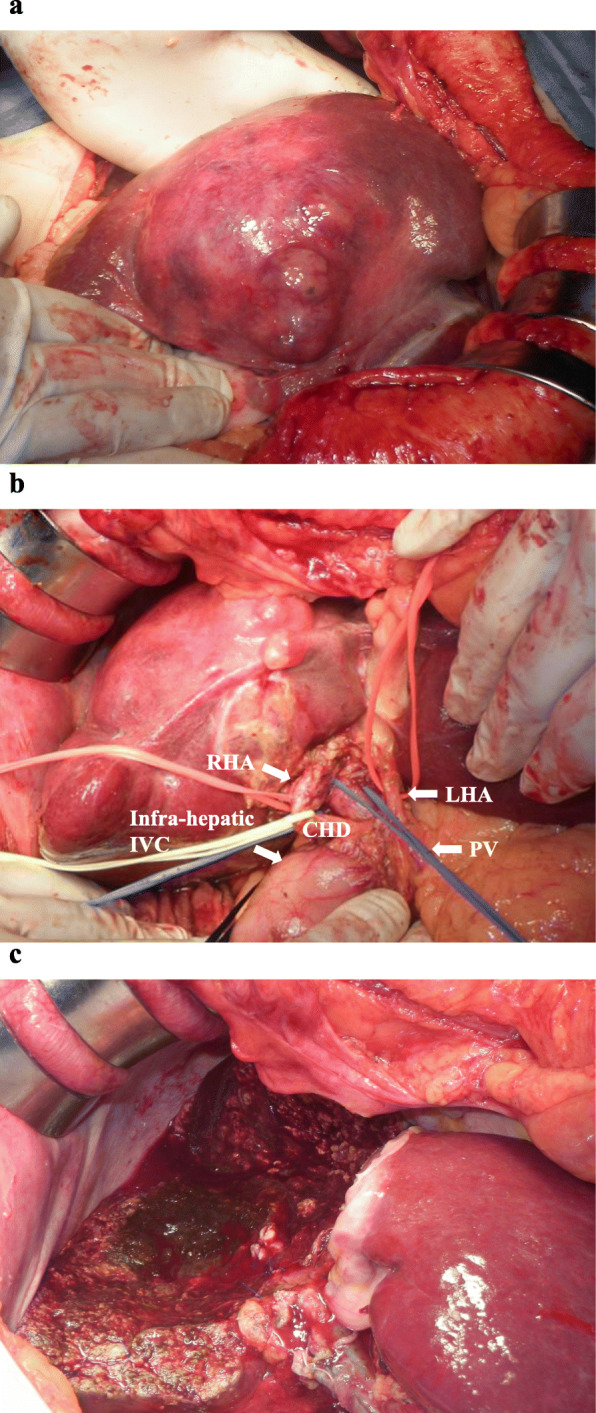
Intra-operative finding. **a** An elastic hard expanding tumor in the segments 4, 5, and ventral site of the segment 8. **b** Hepatic hilum dissection and encircling of the infra-hepatic IVC with a tape. RHA: right hepatic artery; LHA: left hepatic artery; CHD: common hepatic duct; PV: portal vein; IVC: inferior vena cava. **c** Completion of the central hepatectomy without the caudate lobectomy of the liver

**Fig. 3:**
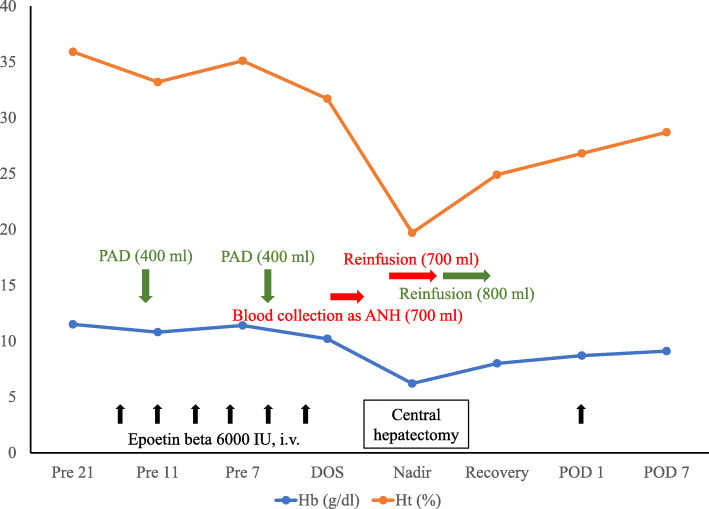
Peri- and intra-operative course of Hb and Ht values with description of PAD and ANH under erythropoietin therapy. Hb: hemoglobin; Ht: hematocrit; PAD: preoperative autologous donation; ANH: acute normovolemic hemodilution; Pre: preoperative day; DOS: day of surgery; Nadir: intraoperative hemoglobin nadir; Recovery: in recovery room

**Fig. 4:**
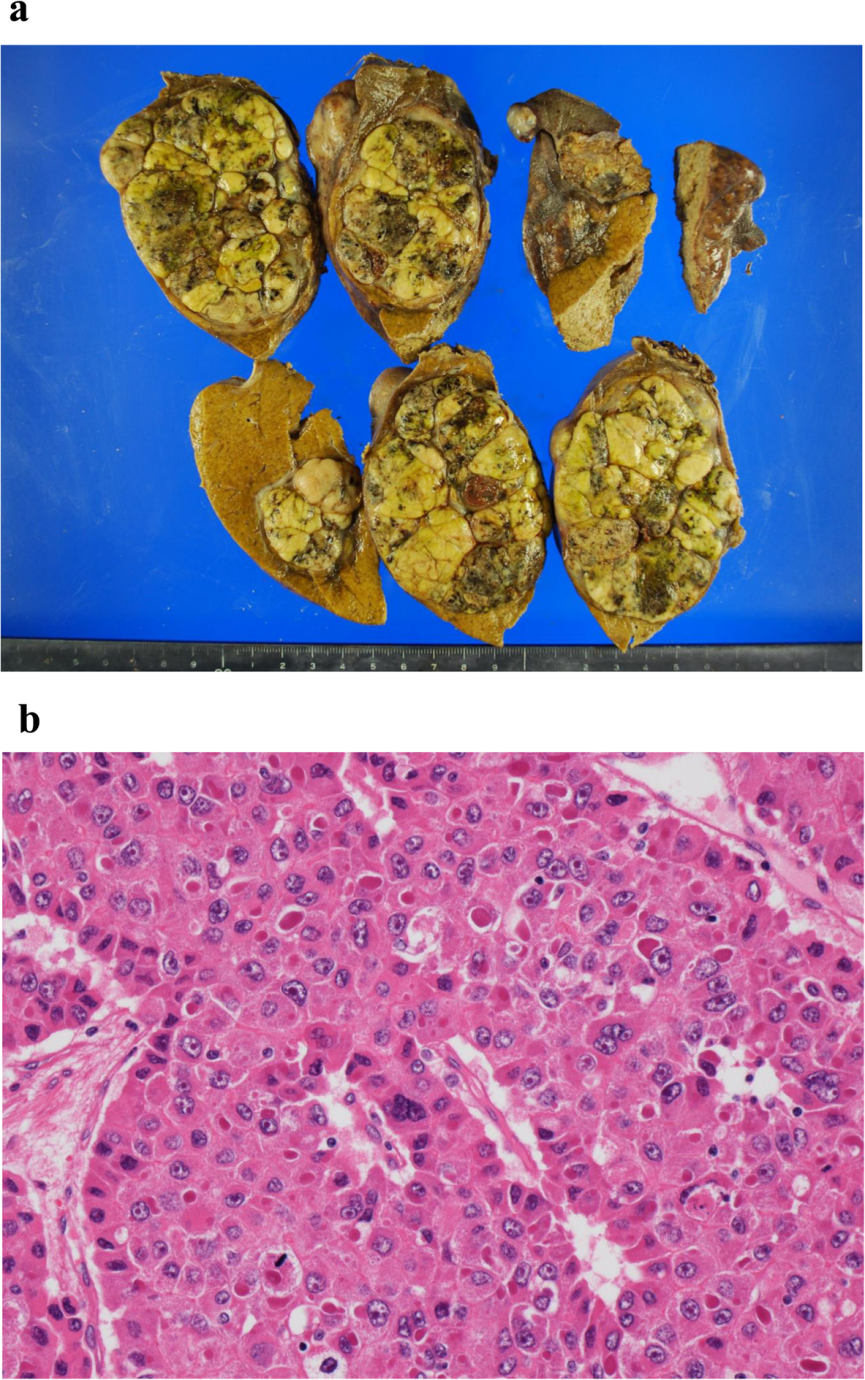
Pathological finding of the specimen. a The tumor macroscopically showed a confluent multinodular type. **b** The tumor was moderately differentiated hepatocellular carcinoma (Hematoxylin and eosin staining)

Several measures were incorporated after the induction of general anesthesia. These included insertion of Swan-Gantz catheter for evaluation of cardiac function for moderate to severe aortic stenosis; insertion of a flexible double lumen catheter for continuous hemodiafiltration (CHDF) in preparation to deal acute HTRs preceded by an unanticipated transfusion; a collection of 700 ml autologous blood as ANH; and a “stand-by” set up of intraoperative cell salvage. Surgery was performed through an inverted T-shaped incision. The tumor was located in the S4, S5, and S8 of the liver (Fig. 2a). First, a cholecystectomy with an insertion of a 6 Fr. tube via a cystic duct for post-hepatectomy bile leakage test was performed, and was followed by the dissection of the hepatic hilum. The middle hepatic artery and the anterior branch of the right hepatic artery originated from the superior mesenteric artery were ligated and divided. Infra-hepatic inferior vena cava (IVC) above the confluence of the left renal vein was encircled by a cotton tape with a tourniquet (Fig. 2b). Mobilization of the right lobe of the liver was performed with the division of the right coronary, triangle, and the hepato-renal ligaments, while the short hepatic veins were not divided. Liver transection was performed with Thunderbeat® (TB) (Olympus Medical Systems Corp., Tokyo, Japan) and a cavitron ultrasonic surgical aspirator (CUSA) along with the Pringle maneuver in cycles of clamp/unclamp time of 15/5 min. After intravenous administration of 100 mg of hydrocortisone, parenchymal transection was initiated just right to the falciform ligament, during which inflow structures of S4 arising off of the hilar plate were ligated and divided. During the transection of parenchyma on this plane, down to the para-caval portion of the caudate lobe, we encountered a longitudinal split injury on the dorsal side of the middle hepatic vein at the confluence of one of the drainage veins from S4B. The liver was transected just left to the right hepatic vein by using TB alone under simultaneous Pringle maneuver and infra-hepatic IVC clamping, while preserving hemostasis with digital compression of the injured portion. The middle hepatic vein was clamped about 2 cm distal from its root, divided, and double ligated at the proximal site. Glissonian pedicles of S5 and S8 was double ligated and divided, respectively, and a CH without caudate lobectomy was performed (Fig. 2c). However, a small portion of S8 and S4B was spared. Frozen sections of the surgical margins revealed negative margins. Hemostasis of the transection line was achieved with suture ligation and soft coagulation. Bile leakage test was performed by using indigocarmine dye. Tachosil^Ⓡ^ was applied to the raw surface and a closed suction drain was placed. The estimated blood loss was 1380 ml without requiring intraoperative cell salvage and an allogenic blood transfusion. The operative time was 260 min. The weight of resected liver specimen was 342 g. The peri- and intra-operative course of Hb and Ht values with description of PAD and ANH under erythropoietin therapy was demonstrated in Fig. 3. Pathologically, the tumor was found to be a moderately differentiated HCC measuring 9.5 × 8.0 cm with negative surgical margins (Fig. 4).

On postoperative day (POD) 1, the Hb level was 8.7 mg/dl and the Ht was 27%. The total bilirubin was 0.7 mg/dl, AST was 621 U/l, ALT was 451 U/l, and PT was 67% (INR 1.27). Anticoagulation therapy was resumed on POD 2 after confirming the absence of bleeding complications. The postoperative course was uneventful except for congestive heart failure classified as grade II by Clavien-Dindo classification [7]. On POD 18, the patient was discharged from the hospital with her Hb level, 9.8 mg/dl; Ht, 32%; total bilirubin, 0.6 mg/dl; AST, 29 U/l; ALT, 40 U/l and PT, 50% (INR: 1.56). She is alive with no sign of recurrence after 45 months after surgery.

## Discussion

In this report, we demonstrated how CH for HCC could be successfully performed in a patient with positive anti-Ge alloantibody without the need for allogeneic blood transfusion. A multidisciplinary team approach tailored with utmost technical adjustments appears to be crucial in this challenging and high-risk surgery.

Preoperative management included PAD [8] facilitated by erythropoietic therapy with recombinant human erythropoietin supplemented by daily administration of iron [9, 10]. The patient underwent 2 times of PAD as often as once a week with 400 ml withdrawal of whole blood until 72 h before surgery [11]. In addition, extensive discussions regarding the natural course of the disease [12], the treatment options [13], the standard surgical strategies, the expected intraoperative blood loss [5], and the prediction and preparation for adverse events in case of incompatible blood transfusion [2] are pivotal. We had three separate meetings and repeatedly discussed all the details with the patient and the team before we agreed to proceed for the surgery.

The intraoperative transfusion management included measures to avoid and at the same time, prepare, for incompatible transfusion; autologous blood collection as ANH immediately prior to operation [14]; setting for blood salvage with a cell saver [15]; insertion of a flexible double lumen catheter for CHDF [16]. ANH is known to be one of the best strategies to prevent perioperative red blood cells (RBCs) transfusion; however, the amount of blood withdrawal, leading hemodilutional coagulopathy and fluid overload remain major concern [14]. The withdrawal volume of whole blood as ANH was determined by the anesthesiological team according to estimation of initial blood volume (65 ml/kg) and initial and target hemoglobin. Thus, 700 ml of blood to be collected was determined by the formula [17]: *V* = EBV × (H_i_–H_f_)/H_av_, where *V* was the volume of blood to be withdrawn (700 ml in this case), EBV was the estimated blood volume of the patient (2860 ml in this case), *H*_i_ was the initial hematocrit before the procedure (32% in this case), *H*_f_ was the target final hematocrit after hemodilution (25% in this case), and *H*_av_ was the average of the *H*_i_ and H_f_ (28.5% in this case). In fact, her Hb level during liver resection immediately after hepatic vein injury dropped up to 6.2 g/dl. Autologous blood reinfusion was also performed under preference by anesthesiologists based on the Hb level, hemodynamics, and the progression status of the operation. We escaped using blood salvage system as many onco-surgeons are reluctant to use the blood for the theoretical risk of tumor dissemination [18]. However, surgeon should decide to use or spare the cell salvage depending on the patients’ condition, as no concrete evidence on the sequelae of the salvaged blood in cancer patients exists. Acute and delayed HTRs by incompatible transfusion in patients with anti-Ge alloantibody have not been well-documented. While there were a few reports documented on successful transfusion of Ge-positive RBCs to patients with an anti-Ge alloantibody [19, 20], Baughn and colleagues reported a case of acute HTRs induced by multiple transfusion [2]. Taking into consideration the possibility of life-threating complications by acute HTRs such as vital shock, renal failure and disseminated intravascular coagulation, we inserted a flexible double lumen catheter and prepared for CHDF along with steroid and immunoglobulin.

The surgical strategy included precise surgical techniques with careful dissection; meticulous hemostasis; anesthesiologist-guided low central venous pressure during liver transection; bloodless liver transection with Pringle maneuver and infra-hepatic IVC clamping; and use of TB with CUSA. The liver transection time from the beginning of the parenchyma resection to the removal of the specimen of liver in the patient was 45 min. With this experience, we have been advocating this procedure (TB liver transection under simultaneous Pringle maneuver and infra-hepatic IVC clamping) for especially high complexity major liver resection [21, 22].

Postoperative management included the continuation of erythropoietic therapy supplemented with the daily administration of iron and reinstitution of anticoagulation therapy. A decline in postoperative serum Hb and Ht levels without active evidence of bleeding until POD 3 after liver resection was reported [23]. Careful observation and continuation of erythropoietic therapy are recommended, even if the patient does not have any obvious symptoms of anemia [9]. Early reinstitution of anticoagulation therapy was also essential in this elderly patient. On POD 2, heparin was restarted after confirming the absence of bleeding complications and was switched to warfarin to prevent thromboembolic complications such as major adverse cardiovascular events, cerebrovascular stroke, pulmonary embolism, or portal vein thrombus [24].

## Conclusions

To our knowledge, this is the first case report of hepatectomy in a patient with anti-Ge alloantibody. A multidisciplinary tailored approach remains crucial in establishing a concrete treatment and management modality. PAD and ANH with perioperative erythropoietic therapy combined with bloodless liver resection should be advocated during major hepatectomy in patients with extremely rare blood type.

## Data Availability

Not applicable

## Abbreviations

Ge: Gerbich
HTRs: Hemolytic transfusion reactions
CH: Central hepatectomy
HCC: Hepatocellular carcinoma
Hb: Hemoglobin
Ht: Hematocrit
AST: Aspartate aminotransferase
ALT: Alanine aminotransferase
PT: Prothrombin time
INR: International normalized ratio
CT: Computed tomography
MRI: Magnetic resonance image
S: Segment
AFP: Alpha-fetoprotein
PIVKA-II: Protein induced by vitamin K absence or antagonist II
HCV: Hepatitis C virus
RLV: Remnant liver volume
SLV: Standard liver volume
PAD: Preoperative autologous donation
ANH: Acute normovolemic hemodilution
CHDF: Continuous hemodiafiltration;
IVC: Inferior vena cava
TB: Thunderbeat^Ⓡ^
CUSA: Cavitron ultrasonic surgical aspirator
POD: Postoperative day

## References

[1] Okubo Y, Yamaguchi H, Seno T, Kikuchi M, Abe S, Ishijima A (1984). The rare red cell phenotype Gerbich negative in Japanese. Transfusion..

[2] Baughn MR, Whitacre P, Lo GS, Pandey S, Lane TA (2011). A mild acute hemolytic transfusion reaction in a patient with alloanti-Ge3: a case report and review of the literature. Transfusion..

[3] Lee MK, Gao F, Strasberg SM (2016). Completion of a Liver Surgery Complexity Score and Classification Based on an International Survey of Experts. J Am Coll Surg.

[4] Lee SY, Sadot E, Chou JF, Gönen M, Kingham TP, Allen PJ (2015). Central hepatectomy versus extended hepatectomy for liver malignancy: a matched cohort comparison. HPB : the official journal of the International Hepato Pancreato Biliary Association.

[5] Chan J, Perini M, Fink M, Nikfarjam M (2018). The outcomes of central hepatectomy versus extended hepatectomy: a systematic review and meta-analysis. HPB : the official journal of the International Hepato Pancreato Biliary Association.

[6] Urata K, Kawasaki S, Matsunami H, Hashikura Y, Ikegami T, Ishizone S (1995). Calculation of child and adult standard liver volume for liver transplantation. Hepatology..

[7] Dindo D, Demartines N, Clavien PA (2004). Classification of surgical complications: a new proposal with evaluation in a cohort of 6336 patients and results of a survey. Ann Surg.

[8] Vassallo R, Goldman M, Germain M, Lozano M (2015). Preoperative Autologous Blood Donation: Waning Indications in an Era of Improved Blood Safety. Transfus Med Rev.

[9] Schälte G, Janz H, Busse J, Jovanovic V, Rossaint R, Kuhlen R. Life-threatening postoperative blood loss in a Jehovah's Witness, treated with high-dose erythropoietin. Br J Anaesth 2005;94(4):442-4. 10.1093/bja/aei068 15653706

[10] Nishida S, Madariaga JR, Santiago S, Quintini C, Palaios E, Gyamfi A (2007). Right trisectionectomy of the liver for intrahepatic cholangiocarcinoma with bile duct invasion in a Jehovah's Witness. J Hepato-Biliary-Pancreat Surg.

[11] Goodnough LT. Autologous blood donation. Crit Care. 2004;8 Suppl 2(Suppl 2):S49-S52. 10.1186/cc2408 PMC3226143 15196325

[12] Choi WM, Yu SJ, Ahn H, Cho H, Cho YY, Lee M (2017). A model to estimate survival in ambulatory patients with hepatocellular carcinoma: Can it predict the natural course of hepatocellular carcinoma?. Dig Liver Dis.

[13] Dank M, Padányi P (2018). Systemic treatment options of primary hepatocellular carcinoma. Magy Onkol.

[14] Saito J, Hirota K (2019). The volume of acute normovolemic hemodilution. Gynecol Oncol Rep.

[15] Zacharias T, Ahlschwede E, Dufour N, Romain F, Theissen-Laval O (2018). Intraoperative cell salvage with autologous transfusion in elective right or repeat hepatectomy: a propensity-score-matched case-control analysis. Can J Surg.

[16] Namikawa A, Shibuya Y, Ouchi H, Takahashi H, Furuto Y (2018). A case of ABO-incompatible blood transfusion treated by plasma exchange therapy and continuous hemodiafiltration. CEN Case Rep.

[17] Balzan S, Gava V. Principles of Hepatic Surgery. Bentham e Books; 2016. p. 126.

[18] Nieder AM, Simon MA, Kim SS, Manoharan M, Soloway MS (2004). Intraoperative cell salvage during radical prostatectomy: a safe technique for Jehovah's Witnesses. Int Braz J Urol.

[19] Hildebrandt M, Hell A, Etzel F, Genth R, Salama A (2000). Determination and Successful Transfusion of Anti-Gerbich-Positive Red Blood Cells in a Patient with a Strongly Reactive Anti-Gerbich Antibody. Infusionsther Transfusionsmed.

[20] Hadley A, Wilkes A, Poole J, Arndt P, Garratty G (1999). A chemiluminescence test for predicting the outcome of transfusing incompatible blood. Transfus Med.

[21] Aryal B, Komokata T, Yasumura H, Kamiimabeppu D, Inoue M, Yoshikawa K (2018). Evaluation of THUNDERBEAT(R) in open liver resection- a single-center experience. BMC Surg.

[22] Komokata T, Aryal B, Tada N, Nuruki K. The high complexity major liver resection by Thunderbeat with the Pringle maneuver and infra-hepatic inferior vena cava clamping. Asian J Surg. 2020. 10.1016/j.asjsur.2020.01.001 31952879

[23] Torzilli G, Gambetti A, Del Fabbro D, Leoni P, Olivari N, Donadon M (2004). Techniques for hepatectomies without blood transfusion, focusing on interpretation of postoperative anemia. Arch Surg.

[24] Haigh PI, Bilimoria KY, DiFronzo LA (2011). Early postoperative outcomes after pancreaticoduodenectomy in the elderly. Arch Surg.

